# Occult HBV Infection May Be Transmitted through Close Contact and Manifest as an Overt Infection

**DOI:** 10.1371/journal.pone.0138552

**Published:** 2015-10-12

**Authors:** Li-Ping Hu, De-Ping Liu, Qin-Yan Chen, Tim J. Harrison, Xiang He, Xue-Yan Wang, Hai Li, Chao Tan, Qing-Li Yang, Kai-Wen Li, Zhong-Liao Fang

**Affiliations:** 1 Guangxi Zhuang Autonomous Region Center for Disease Prevention and Control, Guangxi Key Laboratory for the Prevention and Control of Viral Hepatitis, Nanning, Guangxi 530028, China; 2 School of Preclinical Medicine, Guangxi Medical University, 22 ShuangYong Road, Nanning, Guangxi 530021, China; 3 Chong Zuo Center for Disease Prevention and Control, Lijiang road, Chong Zuo, Guangxi 532200, China; 4 Division of Medicine, UCL Medical School, London, United Kingdom; 5 Guangdong Provincial Institute of Public Health, Guangdong Provincial Center for Disease Control and Prevention, Guangzhou 511430, China; Chiba University, Graduate School of Medicine, JAPAN

## Abstract

The importance of transmission of occult HBV infection (OBI) via transfusion, organ transplantation and hemodialysis has been widely recognized. However, data regarding the transmission of OBI through close contact remain limited. In this study, serum samples were obtained from a child and his parents. The child had received the standard vaccination regimen at birth and produced protective antibody. Sera were tested for HBV serological markers. Nested PCR assays were used to detect HBV DNA and the amplicons were cloned and their sequences subjected to phylogenetic analysis. The results showed that both parents had occult infections while the child had an overt infection. Twelve, eleven and nine clones, from the father, mother and son, respectively, were sequenced. Serotypes adrq+, ayw1, ayw and ayr were found in the father and ayw1, adw2 and adwq+ in the mother; adrq+ was the only serotype in son. Genotype B, subgenotype C2 and a recombinant were identified in the father and genotype B, subgenotype C5 and three recombinants were found in the mother. Subgenotype C2 was the only genotype identified in the child. A phylogenetic tree showed that all of the child’s sequences and most of the father’s sequences clustered together. However, none of mother’s sequences clustered with those of the child. The surface gene from the child and his father had the same amino acid substitution pattern (T118K, T123N and G145A). We concluded that the father was the source of the son’s HBV infection, suggesting that occult HBV infection may be transmitted through close contact and manifest as an overt infection.

## Introduction

Persistent infection with hepatitis B virus (HBV) remains a major global public health problem. Infection with hepatitis B virus may lead to a wide spectrum of liver disease that range, in acute infection, from mild, self-limited to fulminant hepatitis and, in persistent infection, from an asymptomatic carrier state to severe chronic hepatitis, cirrhosis and hepatocellular carcinoma. More than two billion people, one third of the world’s population alive today, have been infected with HBV at some time in their lives and around 240 million of them remain infected (chronic HBsAg carriers) [1]. However, these classes do not include all HBV infections; there is another form of HBV infection, occult HBV infection (OBI). This was first reported by Tabor et al. 36 years ago in a case report of HBV infection following blood transfusion with blood from donors positive for anti-HBc only [2]. OBI is defined by the absence of HBsAg despite the presence of HBV DNA in the liver, blood serum, or peripheral blood mononuclear cells, irrespective of the presence of other hepatitis B viral antibodies and antigens [3].

The prevalence of OBI varies widely across the globe and ranges from 1% to 95% worldwide, depending on the level of endemic disease, the assays used in the studies and the various populations studied [4, 5]. However, it is well known that certain groups of patients are at a much higher risk of having occult HBV infection, regardless of the geographical location, such as those with chronic HCV infection and HIV infection, hemodialysis, liver transplant and hepatocellular carcinoma (HCC) patients and injection drug users [6]. Occult infection may be reactivated, leading to acute and severe forms of classical hepatitis B. The long-term persistence of the virus in the liver may favor the progression of the chronic liver disease to cirrhosis and HCC [7].

One of the major public health problems of occult HBV infection is the potential for transmission. Blood transfusion and liver transplantation remains the major routes of transmission, although the risk of HBV transmission through blood transfusion has decreased following the introduction of sensitive and specific diagnostic assays [8]. In additional, it has also been reported that intrauterine HBV infection is possible in pregnant women who are HBsAg and HBeAg negative [9]. The possibility of horizontal transmission of HBV from individuals with occult infection to close contacts does exist [10]. When occult viruses are transmitted to other individuals, the outcomes in terms of liver disease are the same as those following transmission from overt cases [8].

It has been reported that the prevalence of OBI is higher in HBV endemic areas such as East Asia and lower in low endemic areas such as North America [5]. Guangxi is one of the provinces in China with the highest prevalence of persistent HBV infection, affecting 9.2% of the general population [11]. We reported previously that the prevalence of OBI among family members of children from Long An county, Guangxi who were positive for both HBsAg and anti-HBs after vaccination is 11.5% [12], suggesting that occult HBV infection is common in Guangxi. In this study, we provide evidence of transmission of occult HBV from a family contact to a child who was vaccinated successfully at birth but became infected overtly.

## Materials and Methods

### Study population and sample design

The study subjects were a three member family, a boy and his parents. Serum samples were obtained from the three individuals in April, 2015.

The father is 44 years old. He was given a full course of immunization (10 μg doses of vaccine given at 0, 1 and 6 months) in1994. He was negative for serological markers of HBV infection before vaccination and after the last dose. He was vaccinated again in 1999 according to the same program but the doses were 30 μg, 20 μg and 10 μg, respectively. He remained negative for all of HBV serological markers after the last dose. He became weakly positive for anti-HBc in 2004 and positive for both anti-HBe and anti-HBc in 2008. He was vaccinated again in 2009 with one 60 μg dose. He was positive for anti-HBc only after vaccination.

The woman is also 44 years old and had married in 1999. She was found to be positive for anti-HBs in 1996. She was immunized with a full course of vaccination (10 μg doses of vaccine given at 0, 1 and 6 months) in 2004 when she found to be weakly positive for anti-HBs. She was positive for anti-HBs (298 IU/ml), anti-HBe and anti-HBc in 2008.

The child was born in 2001. He received in time a full course of vaccination (10 μg doses of vaccine given at 0, 1 and 6 months). He was anti-HBs positive with a titer of ≥10 IU/L after the last dose. However, he became positive for HBsAg in 2004. He was positive for HBsAg, anti-HBe and anti-HBc in 2007.

All vaccines used above are yeast-derived recombinant hepatitis B vaccine (National Vaccine and Serum Institute, Beijing, China).

Informed consent in writing was obtained from the parents and that of the child was from the parents in his behalf. The study protocol conforms to the ethical guidelines of the 1975 Declaration of Helsinki and has been approved by the Guangxi Institutional Review Board.

### Serological Testing

Sera were tested for HBsAg/anti-HBs, HBeAg/anti-HBe, anti-HBc and anti-hepatitis C virus (HCV) using enzyme immunoassays (Zhong Shan Biological Technology Company, Limited, Guangzhou, China). Alanine aminotransferase (ALT) levels were determined using a Reitman kit (Sichuan Mike Scientific Technology Company, Limited, Chengdu, China).

### Nested polymerase chain reaction (PCR) for HBV DNA and nucleotide sequencing

DNA was extracted from 85 μl serum by pronase digestion followed by phenol/chloroform extraction. In order to avoid false positive, two regions of the HBV genome were amplified using nested PCR, from PreS1 to the X gene and a smaller region covering the S gene only.

For PreS1 to the X gene, the first round PCR was carried out in a 50 μl reaction using primers LSOB1 (nt 2739–2762, 5'-GGCATTATTTGCATACCCTTTGG-3') and P2 (nt 1823–1806 5'-CCGGAAAGCTTGAGCTCTTCAAAAAGTTGCATGGTGCTGG-3’) [13], with 5 min hot start followed by 30 cycles of 94°C for 30 sec, 50°C for 30 sec, and 72°C for 90 sec. Second round PCR was carried out on 5 μl of the first round products in a 50 μl reaction using primers LSBI1 (nt 2809–2829, 5'-TTGTGGGTCACCATATTCTT-3') and POLSEQ2 (nt1168-1188, 5'-AGCAAACACTTGGCATAGGC-3') and the same amplification protocol as first round.

For the S gene, the first round PCR was carried out in a 50 μl reaction using primers MD14 (nt 418–433, 5'-GCGCTGCAGCTATGCCTCATCTTC-3') and HCO2 (nt 761–776, 5'-GCGAAGCTTGCTGTACAGACTTGG-3'), with 5 min hot start followed by 35 cycles of 94°C for 45 sec, 45°C for 45 sec, and 72°C for 120 sec. The second round PCR was carried out on 5 μl of the first round products in a 50 μl reaction using primersME15(nt 455–470, 5'-GCGCTGCAGCAAGGTATGTTGCCCG-3') and HDO3 (nt 734–748, 5'-GCGAAGCTTCATCATCCATATAGC-3') with 5 min hot start followed by 30 cycles of 94°C for 45 sec, 55°C for 45 sec, and 72°C for 120 sec.

Amplicons from the second round were confirmed by agarose gel electrophoresis and cloned into the vector pUCm-T (The Sangon Biotech (Shanghai, China)). Plasmid DNA was extracted using a SK1191 UNIQ-10 kit (The Sangon Biotech (Shanghai, China)) and the purified DNA was sequenced using a BigDye Terminator V3.1 Cycle Sequencing kit (Applied Biosystems, Foster City, USA) with sequencing primer PSISEQ2F (nt 65–84, 5'-GGCTCCAATTCCGGAACAGC-3') and POLSEQ2. Meanwhile, PreS1/S2 region of HBV from each sample was sequenced directly without cloning using sequencing primer LSBI1 in The Sangon Biotech (Shanghai, China).

### Measurement of Viral loads

Serum HBV DNA concentrations were quantified by real time PCR using commercial reagents (Shanghai ZJ Bio-Tech Co., Ltd. (Shanghai, China)) in an ABI Prism 7500 sequence detection system (Applied Biosystems, Foster City, CA, California, USA), using HBV primers and a dual labeled TaqMan probe, as described previously [14].

### HBV serotyping

Serotypes were determined according to a single amino acid at the following position: adrq- (122 K + 127P + 134 F + 159 V + 160R +177A +178P), adrq + (122 K + 127P + 134 F + 159A +160R + 177 V +178P), adw2 (122 K + 127P + 134 F +159A + 160 K + 177 V +178P), adwq+(122 K + 127 L +134 F + 159A + 160 K + 177 V +178P), ayr (122R + 127P +134 F + 159A + 160R + 177 V +178P), ayw1 (122R +127P + 134 F + 159A + 160 K + 177 V +178P), ayw2 (122R + 127P + 134Y + 159G + 160 K + 177 V +178P), ayw3 (122R + 127 T + 134 F + 159G + 160 K + 177V +178P) [15–17].

### HBV genotyping

HBV genotypes were determined using phylogenies reconstructed on the basis of the complete S region (678 nt) of the viruses. The sequences were aligned to 21 HBV sequences of all known genotypes retrieved from GenBank using Clustal W and visually confirmed with the sequence editor BioEdit [18]. The reference sequences were A1_M57663_Philippines, B1_D23677_Japan, B2_AY217358_China, B2_AF121249_Vietnam, B3_AB033555_Sumatra, B4_AB073835_Vietnam, B5_AB219427_Philippines, B6_DQ463801_Canada, C1_AF458664_China, C2_AY217371_China, C3_X75656_Polynesia, C4_AB048704_Australia, C5_JN827415_Thailand, D1_AF280817_China, E_AB091255_Ivory Coast, F_AY090458_Costa Rica, G_AF160501_USA, H_AY090460_USA, I1_AB231908_Vietnam, I1_FR714504_Longan_China, I2_FJ023664_Laos. Neighbor-Joining trees were reconstructed under the Kimura 2-parameter substitution model with the program MEGA [19]. The reliability of clusters was evaluated using interior branch test with 1000 replicates and the internal nodes with over 95% support were considered reliable.

Sequences that were not determined by phylogenies were genotyped using the NCBI Genotyping Tool (http://www.ncbi.nlm.nih.gov/projects/genotyping/formpage.cgi).

### Identification of MHR mutations and overlapping polymerase mutations

Amino acid substitutions in the major hydrophilic region (MHR; aa 99–169) were originally evaluated using the Genafor/Arevir-geno2pheno drug resistance tool (http://hbv.geno2pheno.org/index.php). Those identified as MHR substitutions by the tool were then aligned to HBV reference sequences (JQ688404, EU410081 and AB776908), which were obtained from GenBank and used to exclude subgenotypes and polymorphisms. The mutations were categorized into general mutations and escape mutations.

## Results

### General information

Successful PCR amplification of two regions of the viral genome was achieved for all three individuals. Both parents have OBI, the father is positive for anti-HBc only and the mother is positive for anti-HBs and anti-HBc. The ALT levels of the parents are normal but that of the child is abnormal. The viral load of the father is the highest and that of the mother is the lowest (Table 1). All three individuals are negative for anti-HCV. Complete PreS1/S2 sequences were obtained for the three individuals; 12, 11 and 9 clones were constructed from the father’s, mother’s and son’s amplicons, respectively. The S-gene was sequenced in both directions, covering the entire MHR (GenBank accession number: KT585753-KT585784)‏.

**Table 1:** Serological characteristics of the study subjects.

Samples	Ages	HBsAg	Anti-HBs	HBeAg	Anti-HBe	Anti-HBc	Viral loads	ALT (IU/ml)
Father	44	**-**	**-**	**-**	**-**	**+**	8×10^5^ IU/ml	<40
Mother	44	**-**	**+**	**-**	**-**	**+**	3.53×10^3^ IU/ml	<40
Son	14	**+**	**-**	**+**	**-**	**+**	5.42×10^5^ IU/ml	170

### Serotypes in different clone sequence

Serotypes adrq+, ayw1, ayw and ayr were predicted from the father’s twelve sequences. Four serotypes, including ayw1, adw2 and adwq+ were predicted from the mother’s sequences. All nine sequences from the son’s sample predicted serotype adrq+ (Table 2). These data suggest that transmission between father and son is possible because they have the same serotype adrq+ and the transmission is from father to son because the father has more serotypes. Transmission between the father and mother also is possible because they share serotype: ayw1. However, transmission between the mother and son is not possible for because they do not share any serotype.

**Table 2:** Serotypes and genotypes predicted from the sequences from each study subject.

Study subject	Number of clones	Serotypes	Genotypes
**Father**			
	9	adrq+	C2
	1	ayw1	B
	1	ayw	B
	1	ayr	Recombinant (B/C)
Total	12		
**Mother**			
	7	adwq+	C5
	1	ayw1	B
	1	ayw1	Recombinant (B/C)
	1	adw2	Recombinant (B/C)
	1	adwq+	Recombinant (C/G)
Total	11		
**Son**			
	9	adrq+	C2
Total	9		

### Genotypes in different clone sequences

**Fig 1:**
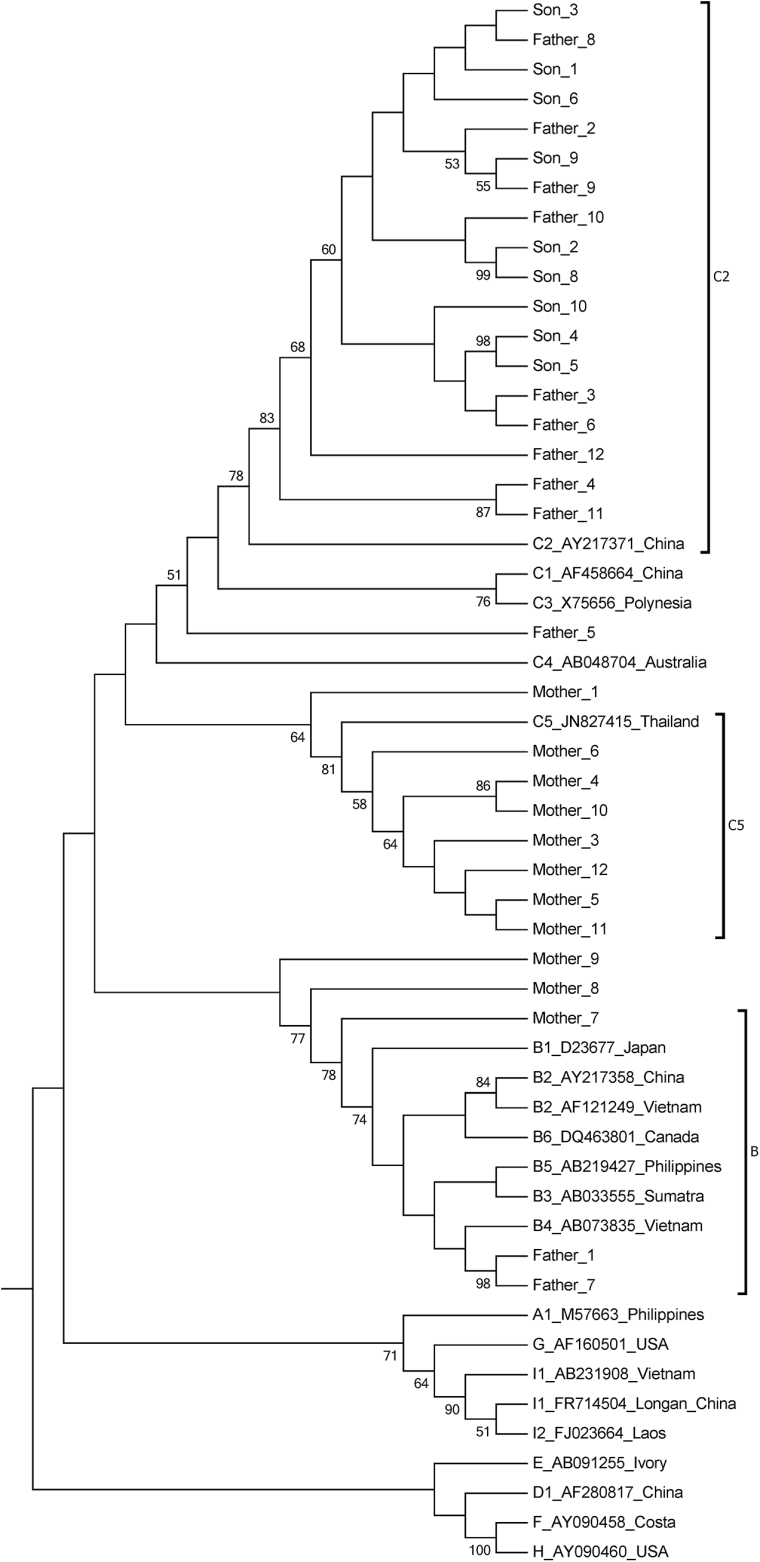
Neighbor-Joining trees. The trees were reconstructed on the basis of the complete S region (678 nt) of the viruses under the Kimura 2-parameter substitution model with the program MEGA [19]. The branch lengths represent the number of substitutions per site. The reliability of clusters was evaluated using the interior branch test with 1000 replicates and the internal nodes with over 95% support are considered reliable.

Using phylogenetic analysis and the NCBI genotyping analysis, three genotypes, subgenotype C2, genotype B and a recombinant were identified from the father’s sequences. Subgenotype C5, genotype B and three recombinants were identified in the mother. Subgenotype C2 was the only genotype identified in the son (Table 2 and Fig 1). The phylogenetic tree shows that all of the son’s sequences and most of the father’s sequences cluster together. However, none of the mother’s sequences cluster with her son’s sequences (Fig 1). Clearly, the transmission was from father to son. There is no evidence of transmission between the mother and son. It is also possible for transmission to have occurred between the parents because some of their sequences cluster together, supported by a 77% bootstrap value.

### Amino acid substitutions within the “a” determinant and MHR

**Fig 2:**
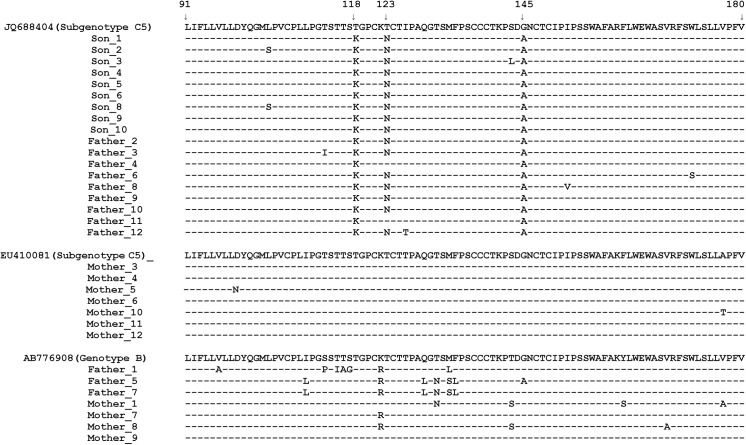
Frequency and distribution of amino acid substitutions in the MHR of HBsAg from each clone.

Amino acid substitutions in the major hydrophilic region predicted from twelve clones of HBV from the father’s sample include T115I, T116A, S117G, T118K, 123N, Q129L, T131N, M133L, M133S, F134L, G145A and I152V. Half of them are located within “a” determinant (aa 124–147). Five of the twelve substitutions, including T118K, T123N, T131N, M133L and N 145A, are associated with antibody escape. The T118K and N145A substitutions may result in vaccine escape. T118K and T123N may result in failure to detect of HBsAg. The 123N and 145A mutations may result from escape from immunoglobin therapy. Seven clones have the same amino acid substitution pattern as that in the son: T118K, T123N and N145A. Two clones have T118K and N145A substitutions, two other clones have T131N and the final clone has M133L (Fig 2).

Amino acid substitutions in the major hydrophilic region predicted from nine clones of HBV from the son’s sample include L104S, T118K, T123N, S143L and G145A. Only two of them are located within the “a” determinant. Except for L104S, all are escape substitutions. All of the nine clones from the son have the same amino acid substitution pattern (T118K, T123N and G145A) as seen in his father. Furthermore, one of the nine sequences has an amino acid substitution at position 143, which may result in vaccine escape and failure to detect HBsAg (Fig 2).

Amino acid substitution mutations in the major hydrophilic region predicted from eleven clones of HBV from the mother’s sample include D99N, T131N, F161S and V168A. Only the T131N mutation causes detection failure and this mutation could be seen in one clone only (Fig 2).

Clearly, the frequency of amino acid substitution mutations within the “a” determinant and MHR is highest in the father’s sample. The next is that from the son. It is possible that there was transmission between father and son because they have the same amino acid substitution pattern. The transmission was from father to son because the father has a more complex pattern of mutations. Transmission between father and mother or mother and son is impossible because they do not share the same amino acid substitution pattern.

### Mutations in the preS1/preS2 region

PreS1 contains 357 bases, encoding 119 amino acids and PreS2 contains 165 bases, encoding 55 amino acids. No deletion was found in either PreS1 or PreS2 from the three samples. No point mutation was found in the initiation codon of preS2 in the three samples.

### The impact of mutations in the S gene on the overlapping polymerase region

Mutations leading to amino acid substitutions in the small S protein may produce amino acid changes in the overlapping polymerase. In this study, there are eleven amino acid substitutions in the overlapping polymerase in all clones from the son’s sample, including R15L, V23I, T38A, T38K, H55Q, S57F, L72P, L77S, H126Q, V191I and H197R. There are twenty-one amino acid substitutions in the overlapping polymerase in all clones from father’s sample, including F46S, R51K, H55Q, H55R, S57F, P109S, N118T, N124R, Y124H, Q125R, H126Q, 127R, N134D, C136R, N139K, Y141F, S143T, H160R, A211T, S213T and Q215H. Amino acid substitutions in mother include T16I, R41S, V44A,N53S, H55R, W58R, N76D, S81T, V103I, G107E, N121I, I122L, N123D, Q125K, H126Y, N134D, N139H, N139Q, Y158H, I163V, F178L, S185N, V207M, Q215L,Y221F, A222T, I224V, G232R. However, none of these is associated with drug resistance.

## Discussion

The major finding in the study is that the son has one serotype (adrq+) only and this was seen in the father but not the mother. The son also has one genotype (subgenotype C2) only and this could also be seen in the father but not the mother. All sequences from the son clustered with that from father in the phylogenetic tree. All of the sequences from the son have the same amino acid substitution pattern in the S protein as that seen in the father. Furthermore, the son was found to be positive for HBsAg when he was tested prior to entrance to kindergarten, suggesting that household contact is the only likely pattern of transmission. These findings provide strong evidence of transmission from father to son. The father shares one serotype and genotype with the mother but not the amino acid substitution pattern in the S protein. Meanwhile, it also is suggested that the mother became infected outside the family. The strength of the study is that detailed medical records are available for the study subjects, which may provide additional evidence of transmission. The weakness of the study is that we did not test HBsAg with different commercial diagnostic kits, which may provide information about the association of amino acid substitutions with detection failure.

The recognized patterns of spread of HBV include perinatal, sexual and parenteral/percutaneous routes. Routes of parenteral transmission include injection drug use, transfusions and dialysis, acupuncture and tattooing; household contact with infected individuals and working in a health-care setting also are risk factors for horizontal transmission [20,21]. Compared to overt HBV infection, the routes of transmission of occult HBV have been studied less. Most of these studies focused on blood transfusion and liver transplantation because these may transmit hepatitis B [8]. In addition, it has also been reported that intrauterine HBV infection is possible in pregnant women with OBI [9].

Occult HBV infection may be common in household contacts of individuals with chronic hepatitis B [22]. A study from India found that sequences from both occult HBV and overt HBV are similar in terms of genotype and surface variants or non-variants (wild-type) and clustered together in the phylogenetic tree. The authors considered the possibility of horizontal transmission of HBV from individuals with occult infection to their contacts [10]. However, this claim is not strong enough because it remains possible that the transmission of HBV was from individuals with overt infection but resulted in occult infection. In our study, with cloning and sequencing, we found that the father has more serotypes and genotypes than that of the son, suggesting that transmission was from father to son.

Immunization with hepatitis B vaccine is the most effective means of preventing acute infection by HBV [23]. However, the titer of vaccine-induced antibody decays exponentially over time, irrespective of the population immunized [24]. It has been claimed that neonatal HBV immunization is efficacious in inducing long-term immunity and cell-mediated immune memory for up to two decades and booster vaccinations are not required [25]. The fact that the child produced protective levels of anti-HBs after immunization but became infected by HBV clearly challenges these findings, suggesting that the monitoring of the level of anti-HBs among vaccinated subjects for booster vaccination is necessary. Our findings also suggested that occult HBV infection may be transmitted through close contact. Therefore, susceptible individuals should be vaccinated against hepatitis B in endemic regions. Nucleic acid testing for OBI is necessary for the staff in some occupations, such as nursery teachers, in regions where HBV is endemic.

Currently, many HBsAg immunoassays use monoclonal antibodies with epitopes directed against the MHR, in particular against the “a” determinant, and amino acid substitution in this region may result in changes to critical epitopes and account for false-negative results in immunoassays [26,27]. The T118K, T123N and N145A substitutions in the MHR have been reported to lead to failure of detection [26, 28– 30]. In this study, ten of twelve clones from the father have these mutations. The remaining two also have detection escape mutations (T131N and M133L) [26, 29]. The father tested negative for HBsAg. All of son’s clones have the same mutations. However, he is positive for HBsAg. It is not clear why the same diagnostic assay produced different test results.

A study from Taiwan showed that most non-responders among anti-HBc positive subjects apparently had occult HBV infection [31]. However, this finding was not supported by a subsequent study from Iran [32]. In our study, the father was negative for all HBV serological markers before and after the first two full courses of vaccination. He became weakly positive for anti-HBc many years later. Then, he was immunized for the third time but remained negative for anti-HBs. Clearly, our data support the result from Taiwan and suggest that nucleic acid testing should be considered for non-responders to exclude OBI, especially in regions where HBV is endemic.

The predominant genotype in Guangxi is genotype C, followed by genotypes B and I (a recombinant) [33]. In the study, both parents are infected with genotype B and C and recombinants (between genotype B and C). In the future, we will determine whether the recombinant sequences are from genotype B and C in the same person or from outside sources, which may provide more information about the occurrence of recombination.
